# A Neglected Point in Medical Economics

**Published:** 1914-12

**Authors:** 


### BUFFALO MEDICAL JOURNAL

**A Monthly Review of Medicine and Surgery**

EDITOR AND PUBLISHER, DR. A. L. BENEDICT, 228 Summer St., corner of Elmwood Ave., Buffalo, (Address for all communications. Please make personal and telephone calls before 1 P. M.)

Third, in age, on the western continent. Independent, but supports professional organization. News limited mainly to Western N. Y. and Penn.

Subscription $2.00 a year; $3.00 for two years in advance. Changes and errors in address or failure or duplication of delivery, should be reported immediately.

Yearly Volume 70. DECEMBER, 1914 No. 5

### A Neglected Point in Medical Economics.

In regard to the generally pessimistic view taken by medical writers, including the editor, when discussing this subject, we are reminded of the observation of a professional humorist that more sympathy would be manifested for the ultimate consumer if he did not so frequently wear silk underclothes. Academically, we heartily agree with the opinion that the physician is not adequately paid. When it comes to reconciling the two commonly ascribed reasons for the underpayment: that there is too much competition, so that the average physician cannot secure sufficient employment, and that the physician ought to be paid more because he is overworked, we find ourselves lacking in the requisite logical skill.

In common with other professions in which the individual simply sells his labor, and with small businesses generally, it is only rarely that medicine leads to anything like wealth. In fact, great wealth can only be derived from the exploitation of other men's labor on a large scale or by securing profits from an enormous number of purchasers, thus imitating the taxing function of government. Of course, we refer to wealth earned or, at least obtained by an individual in the pursuit of his occupation, not to inherited or married wealth or to wealth derived from some sudden and, so far as the individual is concerned, purely accidental and abnormally rapid rise in value of some property supposedly of small value.

Everyone, of any common sense, selecting medicine as a career, must realize that it has its financial limitations; also that it usually requires three or four years before a practice can be built up in a city to the point of self-support. So far as we can judge, viewing the matter either from a personal or a general standpoint, the physician after having passed the practically inevitable period of establishing a practice, fares about as well as any one with whom he can reasonably compare himself. Consider, for example, your school mates, your relatives, the majority of the men whom you meet socially and in business ways, and discount the cases in which inherited wealth, influence, marked and recognized ability, afforded opportunities which you yourself did not possess. In our personal experience, we can recall only one instance of signal success in a school mate in the sense of rising from poverty to wealth beyond the moderate degree of comfort generally attained by medical men. We can recall only three similar instances in relatives; in one of these, there was not only unusual ability but an early and wealthy marriage; in another, wealth came late in life from an invention; in another, it came only after a life-time of frugality without marriage. Even in a large general acquaintance, marked financial success has occurred only in a small percentage of cases and almost exclusively in occupations which afford opportunities closed to the medical profession.

These possible advantages of other occupations are offset by disadvantages noted in quite as large a percentage of cases. We physicians understand the trying periods when collections are slow and practice dull but let us contrast such times with the actual loss of salaried position by the acquaintance who, as a teacher, clergyman, newspaper man, engineer or bookkeeper, was so far above our own earning capacity up to the completion of our medical studies, internship and first few years of practice. Physicians fail from alcoholism or other drug addiction, gross negligence and lack of adaptation to the chosen calling. But who ever heard of a physician, after he had once established a practice, failing from extrinsic causes such as, for instance, caused a dozen firms to discontinue business in a single block in Buffalo in the last three years? We do not, of course, allude to bankruptcy from speculation or engaging in other business or from excess of expenditures, but to the failing of the physician's business, itself. Another, very favorable economic item to the credit of the medical career is that the loss of employment due to advancing years, or failure due to the same cause and preventing adaptation to changing business conditions or weakening the judgment, operates very gradually in the case of the physician. The old man in medicine may realize that his practice is not as large as formerly, that his younger colleagues and more critical patients regard him as an old fogy, that he is getting a little rusty, that his hearing is failing him in auscultation or that in other ways he is physically declining and must abandon certain professional demands, but there is no sudden discharge to make room for a younger man and no futile seeking for other work, no crash of his practice because he has made a blunder in its management. Almost the only sudden reminders of approaching age that the physician need fear along these lines are outside the domain of practice in the strict sense and apply to positions of power and honor which he has monopolized to the exclusion of competitors and from which he will be forced as other influences exceed in strength those by which he has held them.

It is very easy to compare our own sad lot with that of the millionaire business man but, if we reflect candidly on our boyhood, most of us were in such a position that we estimated that our chances of success in a business career were not much greater then of becoming president. This judgment is sustained by our observation of the majority of our associates who engaged in any kind of business that might conceivably lead to such a degree of financial success. It is easy, too, to compare ourselves with the petted and pampered minister to a rich congregation but he is no better off than some of our more fortunate professional colleagues and, if we had possessed the tact, eloquence and self control necessary to striking success in the ministry, we would probably be in the same relative position in the medical profession. The same is true of law, various executive fields of labor and the conduct of businesses of less than the greatest magnitude. It is far fairer to rate ourselves in our own profession first, and then to compare ourselves with men of approximately the same rank in other professions, the government services, medical or otherwise, in small independent businesses, or occupying the higher grades of salaried positions. All things considered, we do not believe that medicine suffers in any such comparison, especially if each physician compares himself with the average of his own personal, boyhood associates.

In brief, let us keep the tenth commandment.

